# Single-cell RNA sequencing of the retina in a model of retinitis pigmentosa reveals early responses to degeneration in rods and cones

**DOI:** 10.1186/s12915-022-01280-9

**Published:** 2022-04-12

**Authors:** Duygu Karademir, Vyara Todorova, Lynn J. A. Ebner, Marijana Samardzija, Christian Grimm

**Affiliations:** 1 Laboratory for Retinal Cell Biology, Department of Ophthalmology, University Hospital Zurich, University of Zurich, Zurich, Switzerland; 2 Zurich Center for Integrative Human Physiology, University of Zurich, Zurich, Switzerland; 3 Neuroscience Center Zurich, University of Zurich, Zurich, Switzerland

**Keywords:** Single-cell RNA sequencing, Retina, Retinal degeneration, Photoreceptors, Retinitis pigmentosa

## Abstract

**Background:**

In inherited retinal disorders such as retinitis pigmentosa (RP), rod photoreceptor-specific mutations cause primary rod degeneration that is followed by secondary cone death and loss of high-acuity vision. Mechanistic studies of retinal degeneration are challenging because of retinal heterogeneity. Moreover, the detection of early cone responses to rod death is especially difficult due to the paucity of cones in the retina. To resolve heterogeneity in the degenerating retina and investigate events in both types of photoreceptors during primary rod degeneration, we utilized droplet-based single-cell RNA sequencing in an RP mouse model, *rd10*.

**Results:**

Using trajectory analysis, we defined two consecutive phases of rod degeneration at P21, characterized by the early transient upregulation of *Egr1* and the later induction of *Cebpd*. EGR1 was the transcription factor most significantly associated with the promoters of differentially regulated genes in *Egr1-*positive rods in silico. Silencing *Egr1* affected the expression levels of two of these genes in vitro. Degenerating rods exhibited changes associated with metabolism, neuroprotection, and modifications to synapses and microtubules. *Egr1* was also the most strongly upregulated transcript in cones. Its upregulation in cones accompanied potential early respiratory dysfunction and changes in signaling pathways. The expression pattern of EGR1 in the retina was dynamic during degeneration, with a transient increase of EGR1 immunoreactivity in both rods and cones during the early stages of their degenerative processes.

**Conclusion:**

Our results identify early and late changes in degenerating *rd10* rod photoreceptors and reveal early responses to rod degeneration in cones not expressing the disease-causing mutation, pointing to mechanisms relevant for secondary cone degeneration. In addition, our data implicate EGR1 as a potential key regulator of early degenerative events in rods and cones, providing a potential broad target for modulating photoreceptor degeneration.

**Supplementary Information:**

The online version contains supplementary material available at 10.1186/s12915-022-01280-9.

## Background

The degeneration of light-sensitive rod and cone photoreceptor cells underlies various forms of inherited retinal disorders (IRDs), including retinitis pigmentosa (RP), the primary cause of blindness among children and working-age adults [1, 2]. Over 100 genomic loci are implicated in RP [3], a progressive and heterogeneous group of disorders often characterized by night blindness due to primary rod photoreceptor death, followed by the loss of high-acuity color vision upon a secondary wave of cone photoreceptor death, leading to complete blindness [1].

Due to the innate heterogeneity of the retina, animal models that recapitulate aspects of the disease while preserving the differences among retinal cells are essential for studying IRDs. The *rd10* mouse is an RP model frequently used for therapeutic studies since it exhibits a later onset and slower progression of retinal degeneration than other similar models (e.g., *rd1),* resembling the human autosomal recessive form of the disorder more closely [4].

The *rd10* mouse harbors a spontaneous, autosomal missense mutation in the rod-specific beta-subunit of the phosphodiesterase gene (*Pde6b*). PDE6 hydrolyses cyclic GMP (cGMP) upon absorption of light by rhodopsin, decreasing the open state probability of cGMP-gated Na^+^ and Ca^2+^ channels and hyperpolarizing the photoreceptor cell. The *rd10* mutation causes the destabilization and mislocalization of PDE6B in rods, causing an increase in intracellular cGMP and Ca^2+^ levels [5]. This leads to a primary wave of rod photoreceptor degeneration peaking around postnatal day (P) 21 [6, 7]. Even though cones do not express *Pde6b*, they are indirectly affected by rod degeneration and die in a secondary wave of cone death starting at around P40 [7, 8]. Various consequences of rod death may contribute to the secondary degeneration of cones. The loss of trophic factors secreted by healthy rods, such as rod-derived cone viability factor [9], may lead to an imbalance of glucose metabolism in cones [10], resulting in a slow mode of cell death [11]. In addition, the loss of oxygen-consuming rods in the degenerating retina may impose hyperoxia on cones, causing increased oxidative stress [12]. Despite the importance of cones to vision, studies of cone-specific responses to rod degeneration are hindered by the relatively low numbers of cones in the retina [13, 14]. Therefore, it remains unclear which early effects are induced in cones at the peak of rod degeneration, and how these may initiate or contribute to the events leading to their eventual degeneration.

Transcriptomic analyses of RP mouse models via microarrays [15, 16] and whole-retina RNA sequencing [17, 18] have been instrumental in furthering our understanding of RP, especially regarding the pathophysiological mechanisms involved in retinal degeneration. However, such techniques cannot differentiate between individual cells, potentially missing critical transcriptional changes restricted to specific cell types or subpopulations of cells in different stages of degeneration. Therefore, techniques with higher resolution are needed to overcome cellular heterogeneity. Recent advances in droplet-based single-cell RNA sequencing (scRNAseq) have enabled the detailed characterization of retinal cell types in mouse [14] and human retinas and organoids [19–21], as well as the elucidation of signaling events and critical factors controlling injury responses in microglia [22] and Müller glia [23], in addition to those regulating distinct phases of retinal development [24] at a single-cell resolution.

Here, we analyzed the degenerative process in the *rd10* mouse retina at the single-cell level. Using the 10X Genomics platform, we investigated transcriptomic changes in *rd10* rods with progressing degeneration using trajectory analysis. We also identified cone responses to rod degeneration at P21, which involves the long-term activation of EGR1 (early growth response 1), an immediate-early gene.

## Results

### Droplet sequencing of *rd10* and wild type retinas

To obtain transcriptomes of individual cells during retinal degeneration, we collected retinas from *rd10* and C57BL/6 wild-type control mice at P21, a time point that falls within the primary wave of rod photoreceptor degeneration in *rd10* mice [8]. A male and a female littermate were sequenced for each genotype to ensure equal representation of sexes in the analysis. To avoid potential confounding effects of optical coherence tomography (OCT) on retinal transcriptomes, we confirmed the increased reflectivity of the degenerating outer retina in *rd10* animals with OCT in littermates not used for sequencing (Additional file 1: Fig. S1).

**Fig. 1:**
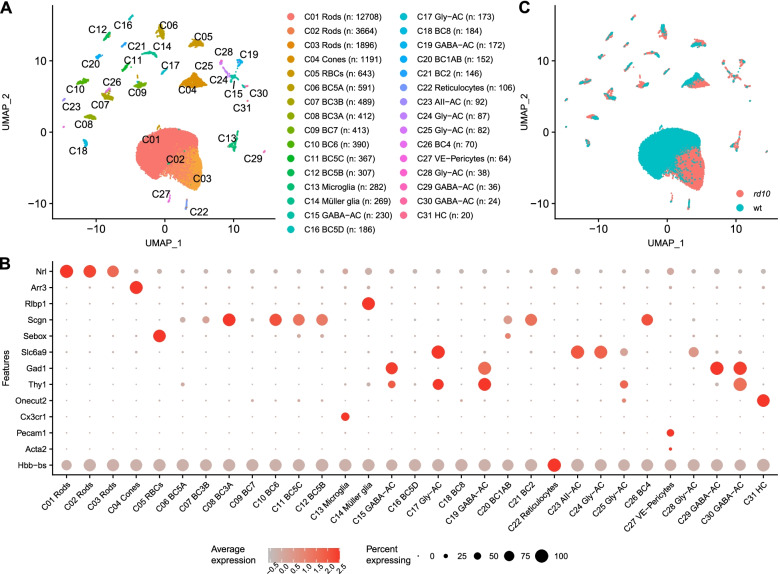
Single-cell transcriptomes from *rd10* and wild-type retinas at P21. **A** Two-dimensional UMAP plot of single cells, colored by cluster identity. n: number of cells. **B** Dot plot representing the expression levels of selected retinal cell type marker genes in each cluster. The color of each dot represents the gene’s expression level across the cluster (average expression), while the size of each dot corresponds to the percentage of cells in the cluster expressing the gene (percent expressing). **C** Two-dimensional UMAP plot of single cells, colored by the genotype of each individual cell. RBC: rod bipolar cells. BC, bipolar cells; AC, amacrine cells; Gly, glycinergic; VE, vascular endothelial cells; HC, horizontal cells

After alignment and demultiplexing with the Cell Ranger pipeline [25], quality control with the Seurat package [26], and a marker gene co-expression-based doublet filtering algorithm [27], we retained 27,374 cells for downstream analyses. Using shared-nearest neighbor embedding and modularity optimization, followed by additional quality control measures (see Methods and Additional file 2: Fig. S2), we filtered our dataset to 25’484 single cells grouped into 31 clusters (Fig. 1A), with 1169 median genes and 2001.5 median unique molecular identifiers (UMIs) per cell. Clusters were annotated using known marker genes [23, 28] (Fig. 1B, Additional file 3). We identified microglia, Müller glia, and all types of retinal neurons except ganglion cells (Fig. 1A, B), which might not have tolerated the dissociation and capture protocols used in our experiments.

While clusters generally included cells from both wild-type and *rd10* retinas, three rod clusters (C01-03) contained almost exclusively either wild-type (C01) or *rd10* (C02-03) cells (Fig. 1C, Table 1). The specificity of the effect to rod clusters excluded a batch effect and instead suggested that wild type and *rd10* rods had distinct transcriptomic profiles (see below and Methods).

**Table 1:** Number of cells per sample

**ID**	**C01**	**C02**	**C03**	**C04**	**C05**	**C06**	**C07**	**C08**	**C09**	**C10**	**C11**	**C12**	**C13**	**C14**	**C15**	**C16**
rd_f	229	989	837	259	196	194	144	129	123	133	89	94	119	46	175	66
rd_m	311	2521	1020	558	329	275	236	181	207	184	205	166	131	160	26	77
wt_f	5429	63	19	125	20	43	22	36	22	26	15	8	12	15	11	18
wt_m	6739	91	20	249	98	79	87	66	61	47	58	39	20	48	18	25
**ID**	**C17**	**C18**	**C19**	**C20**	**C21**	**C22**	**C23**	**C24**	**C25**	**C26**	**C27**	**C28**	**C29**	**C30**	**C31**	**∑**
rd_f	111	52	85	103	103	35	74	79	71	53	16	38	32	22	11	4707
rd_m	44	90	51	19	32	55	6	4	4	10	31	0	2	1	7	6943
wt_f	10	19	23	11	4	3	3	1	6	2	3	0	1	1	0	5971
wt_m	8	23	13	19	7	13	9	3	1	5	14	0	1	0	2	7863

*∑* total in sample, *rd rd10*, *wt* wild type, *f* female, *m* male

### Transcriptomic analysis of rod photoreceptors points to *Egr1* as a critical regulator of early degenerative events

Preceding note: to increase readability, we do not define all gene abbreviations and refer to Ensembl [29] for detailed information on genes of interest.

**Fig. 2:**
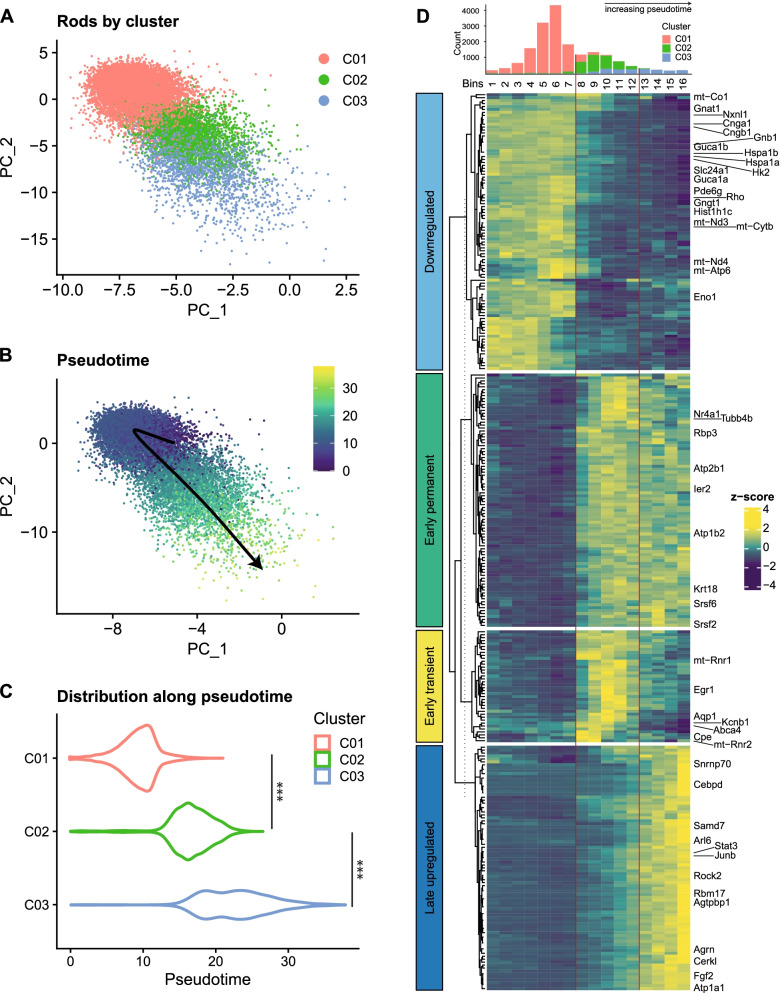
Pseudotime analysis of rod transcriptomes. Rod cells on the recalculated principal component analysis plot were colored according to **A** cluster identity or **B** pseudotime values (alongside the single principal curve). **C** Violin plots of pseudotime values in each rod cluster. Significance of pseudotime differences in each cluster was tested with one-way ANOVA and Tukey’s range test. ***p-value < 2e−16. **D** Rods were grouped into 16 bins according to their pseudotime values. Top: Histogram for rod cell counts from each cluster in each pseudotime bin (*x*-axis)). Bottom: Differential gene expression analyses between clusters adjacent in pseudotime were used to build differentially expressed (DE) gene sets (adjusted *p*-value < 0.05). For each DE gene, average scaled expression (*z*-score) values in each pseudotime bin were used to cluster genes according to their pseudotemporal expression pattern (k-means clustering, *k* = 4). Vertical red bars mark positions in pseudotime where cells’ cluster identities change

Rod degeneration is the primary event in most IRDs and leads to the secondary loss of cones. For an in-depth analysis of rod transcriptomes during their degenerative process, we analyzed the 18,268 rod photoreceptors in clusters C01-C03 (Fig. 2A). Rod degeneration in the *rd10* model exhibits a slow progression [4, 6, 8] with a subtle center-to-periphery gradient [30], suggesting heterogeneity among degenerating *rd10* rods in the same retina, with cells simultaneously at different stages of degeneration. To resolve this heterogeneity in our rod dataset, we used the Slingshot package, which constructs one-dimensional trajectories that connect cells at different stages of continuous processes (“pseudotime”) [31]. We retrieved a single lineage that started from the predominantly wild-type cluster C01, followed sequentially by the predominantly *rd10* clusters C02 and C03 (Fig. 2B, C), with significantly different pseudotime values between the clusters (Fig. 2C). This trajectory indicated that *rd10* clusters C02 and C03 comprised rods at different stages of degeneration, with C03 cells further along the process (“late degeneration”) than C02 cells (“early degeneration”).

To study potential transcriptomic changes during rod degeneration, we restricted our gene set by performing pairwise differential expression (DE) analysis between clusters adjacent in pseudotime: C01-C02 (“Early DE analysis”: Additional file 4) and C02-C03 (“Late DE analysis”: Additional file 5). We then investigated the pseudotemporal behavior of all significant DE transcripts from the two analyses in binned pseudotime (Fig. 2D—bar graph) and defined four major DE patterns with k-means clustering [32]: (1) downregulation, primarily at the onset of C01-C02 transition (potential early degeneration); (2) early permanent upregulation, in which expression was induced early and remained elevated; (3) early transient upregulation, in which expression levels peaked during early pseudotime and returned to near baseline later; and (4) late upregulation, in which expression peaked late in pseudotime (potential late degeneration) (Fig. 2D—heatmap, Additional file 6).

At the potential onset of degeneration, 155 transcripts were significantly upregulated and 72 were downregulated in C02 *rd10* rods compared to wild-type C01 rods (early DE analysis, |logFC| > 0.25, adjusted *p*-value < 0.05, Additional file 4). Various transcripts linked to cellular stress were induced in this stage (Fig. 2D, pseudotime bins 8-12), which was further characterized by the activation of transcription factors that belong to the class of immediate-early genes (*Egr1*, *Nr4a1*, *Ier2*). *Egr1* was the most highly (but transiently) upregulated transcript in early DE analysis (Fig. 2D, Additional file 4). We noted the transient upregulation of several factors potentially involved in the regulation of water (*Aqp1*), ion (*Kcnb1*, *Slc17a7*, *Slc12a5* [permanently upregulated]) and, notably, lipid/retinoid metabolism (*Abca4*, *Sdc4*) and homeostasis (Fig. 2D, Additional file 4). Importantly, both the Ca^2+^ extruder PMCA1 (*Atp2b1*) expressed in mouse photoreceptors [33] as well as the calcium signaling mediators *Camk2g* and *Pcp4* were upregulated early and remained high, indicating an attempt to counteract the increase in intracellular Ca^2+^ levels due to the *rd10* mutation [5] (Fig. 2D, Additional file 4). Furthermore, signaling mediators like *Akap11* and *Mapk8* and several transcripts associated with microtubules (*Tubb5b*, *Arhgef2*, *Agtpbp1*), synaptic structure and transmission (*Cpe*, *Snap25*, *Slc1a2*, *Slc17a7*, *Slc12a5*) as well as anti-apoptotic roles (*Gadd45b* [34], *Krt18* [35]) were upregulated in early degeneration (Fig. 2D, Additional file 4).

Meanwhile, early degenerative *rd10* rods exhibited downregulation of the linker histone H1 variant *Hist1h1c*, followed by *Hspa1a* (HSP70-1) and *Hspa1b* (HSP70-2) (Additional file 4). HIST1H1C has been associated with increased stress and autophagy in diabetic mouse retinas [36], while members of the heat shock protein 70 family are involved in retinal neuroprotection [37]. Similar to models of induced retinal degeneration [38], multiple transcripts involved in phototransduction (*Pde6g*, *Cnga1-b1*, *Guca1a-1b*) decreased after the onset of degeneration in *rd10* rods (Fig. 2D, Additional file 4). In addition, we noted the early downregulation of rod-derived cone viability factor *Nxnl1* [10] and transcripts encoding glycolytic enzymes, particularly hexokinase 2 (*Hk2*), which mediates aerobic glycolysis in photoreceptors and is involved in survival upon stress and aging [39, 40], and enolase-1 (*Eno1*). This suggests an early functional and metabolic disruption in *rd10* rods.

Further along the degenerative process, C03 rods (Fig. 2C) had 78 transcripts upregulated and 39 downregulated compared to C02 rods (late DE analysis, |logFC| > 0.25, adjusted *p*-value < 0.05, Additional file 5). In these cells, the most highly upregulated transcript was *Cebpd* (Additional file 5), a stress-response transcription factor that was shown to be induced in the *rd10* retina [8] but whose cell-type localization was not described. Another strongly regulated transcription factor was *Junb*, an AP-1 component found at increased levels in the light-damaged retina [41] (Additional file 5). These changes were paralleled by a decrease in *Egr1* (Fig. 2D, bins 13-16). Many transcripts associated with survival, specifically with leukemia inhibitory factor-mediated endogenous retinal neuroprotection in models of inherited [42] and induced [43] degeneration (*Fgf2*, *Edn2*, *Stat3*, *Socs3*), were strongly upregulated in these cells (Additional file 5). Concomitantly, levels of transcripts with potential anti-apoptotic functions (*Gadd45b* [34], *Clu* [44], *Cerkl* [45]) (Fig. 2D), were increased (Additional file 5). Furthermore, we observed the continued increase in tubulin deglutamylase *Agtpbp1* and the upregulation of *Arl6*, which is critical in protein trafficking to the connecting cilium [46]. Together with the early upregulation of microtubule-associated transcripts, this may indicate progressive changes to the photoreceptor outer segments (Additional file 4). Potential changes also to synaptic structures were suggested by the upregulation of *Rock2* [47, 48] and *Agrn*, as well as a decrease in VGLUT1 (*Slc17a7*) (Additional file 5).

In late degeneration, the levels of phototransduction mediators (e.g., *Rho*, *Gnb1*, *Gnat1*) decreased further in *rd10* rods (Fig. 2D, Additional file 5) and the potential metabolic disruption observed already in early degeneration was followed by the significant downregulation of genes involved in oxidative phosphorylation in late degenerative rods (Additional file 5). Especially affected were the mitochondrial respiratory complex I NADH dehydrogenase (*mt-Nd1-5*), a crucial subunit of complex III coenzyme Q-cytochrome c reductase (*mt-Cytb*) [49], and complex IV cytochrome c oxidase (*mt-Co1-3*), pointing to mitochondrial respiratory dysfunction at the late degenerative stage (Fig. 2D, Additional file 5).

Several genes connected to retinal degeneration in humans were differentially regulated in degenerating mouse rods, including the downregulation of *Fam161a* (RP [50]) and the upregulation of *Cerkl* (RP [45]), *Arl6* (Bardet-Biedl syndrome [51]), and *Samd7* (RP [52]), a CRX-regulated transcriptional repressor [53] which is essential for rod photoreceptor identity [54] and a paralog of the RP disease gene *SAMD11* [55]. Differential regulation of these genes in mouse rods may additionally validate the *rd10* mouse as a valuable RP model.

In addition to pairwise DE analyses, we utilized the tradeSeq package [56] to test the association of gene expression changes to pseudotime. All but 7 significant DE transcripts as determined by pairwise DE analyses had a statistically significant correlation to pseudotime (Additional file 7), further supporting the relationship of these genes to potential damage progression.

Since the most highly upregulated transcripts in the two distinct stages of rod degeneration encode for transcription factors EGR1 (early) and CEBPD (late), we asked whether they could be the main drivers of transcriptomic changes within their associated stages. To explore this possibility, we performed an in silico promoter scan using Enrichr (TRANSFAC and JASPAR PWMs [position weight matrices]) [57] and searched for exact matches to known transcription factor motifs in a − 2000:+ 500 base range of the transcriptional start site of significantly differentially expressed genes (see above). EGR1 was the transcription factor most strongly associated with the promoters of early degeneration DE genes, based on the Enrichr combined score (Additional file 8). No significant association could be made for late degeneration DE genes (Additional file 9). To validate the in silico analysis, we directly tested whether EGR1 is involved in regulating the expression of *Gadd45b* and *Agtpbp1*, two of the genes predicted to be EGR1 targets (Additional file 8). Using small interfering RNA (siRNA), we downregulated the expression of *Egr1* in photoreceptor-like 661W cells [58]. While the expression of *Apoe*, a gene not predicted to be a target for EGR1, was not affected, the levels of *Gadd45b* and *Agtpbp1* increased significantly upon siRNA-mediated knockdown of *Egr1* (Additional file 10: Fig. S3), suggesting that EGR1 might function as a repressor of these two genes, an activity that was ascribed to EGR1 already by others [59, 60]. This potential regulation is particularly interesting, as we found the levels of both transcripts, especially *Agtpbp1*, to increase further in rods with progressing degeneration, when *Egr1* expression was lost (Fig. 2D).

### The cone response to rod degeneration at P21

**Fig. 3:**
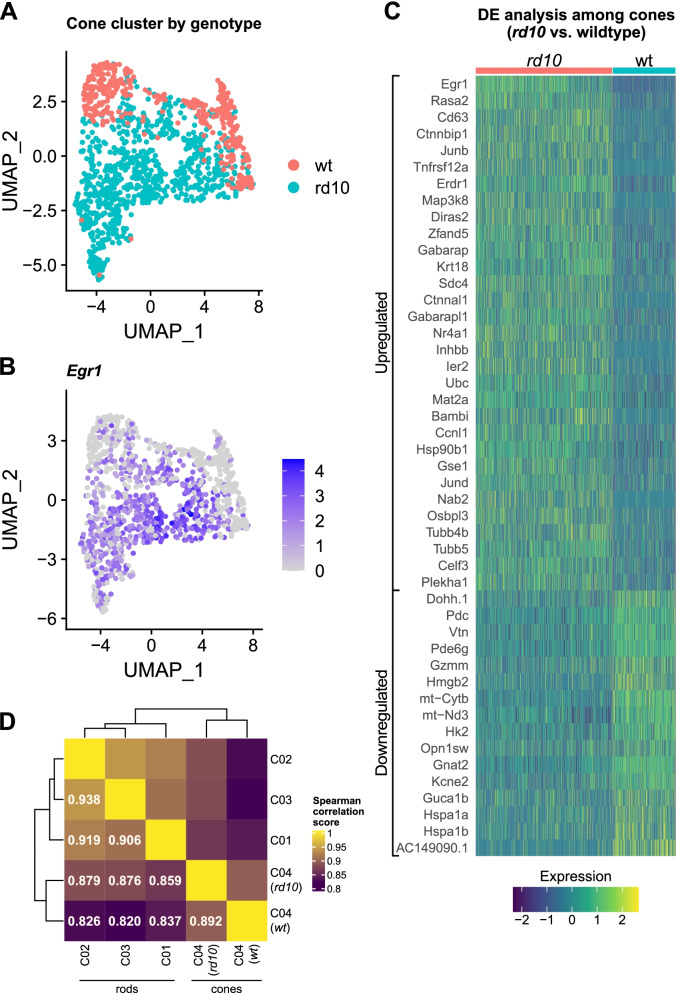
Response of cones to rod degeneration at P21. Two-dimensional UMAP plot of cluster C04 containing cone photoreceptors, colored according to **A** genotype or **B** normalized expression levels of *Egr1*. **C** Dot plot of all differentially expressed genes with absolute average log fold change > 0.5 and adjusted *p*-value < 0.05 among *rd10* and wild-type cones. Heatmap depicts scaled expression values for individual cells. Genes were sorted on the *y*-axis based on average log fold change values. **D** Spearman’s correlation scores for average normalized gene expression between rod clusters C01-03 and cone cluster C04. Cone cluster C04 was separated into two groups according to genotype. 5000 most variable features in the dataset were used for calculating scores. wt, wild type

Even though the mutant *Pde6b* gene that leads to rod degeneration in *rd10* mice is not expressed in cones, cones respond to the loss of rods and degenerate in a second wave of cell death. To address early mechanisms in cones that may lead to this secondary cone cell death, we analyzed the transcriptomes of the 1190 cone cells in cluster C04 (Fig. 1A, B). Differential gene expression analysis between *rd10* and wild type cones in this cluster (Fig. 3A) returned 245 upregulated and 87 downregulated transcripts (|logFC| > 0.25, adjusted *p*-value < 0.05; Additional file 11) and revealed that *Egr1* was the most highly upregulated gene in *rd10* cones (Fig. 3B, C), similar to *rd10* rods early in degeneration (Fig. 2D).

Among upregulated transcripts in *rd10* cones, we noted other immediate-early genes (*Nr4a1*, *Junb*, *Ier2*) and various signaling mediators (e.g., *Inhbb*, *Bambi* [TGF-beta signaling], *Tnfrsf12a* [TNF signaling], *Ctnnbip1* [beta-catenin signaling], *Map3k8*) (Fig. 3C, Additional file 11). Since glucocorticoid receptor activation is neuroprotective against light damage [61], it is interesting that glucocorticoid signaling regulators (*Ncoa6* [62], *Calr* [63, 64], *Arid1a* [65]) were upregulated in cones (Additional file 11). In addition, ER stress and unfolded protein response (*Foxred2*, *Xbp1*, *Ddit3*, *Eif2ak3*, *Pdia6*, *Hsp90b1*) as well as autophagy-related transcripts (*Gabarap*, *Gabarapl1*, and a component of the BRAF35-HDAC complex [*Gse1*]) were expressed at higher levels in *rd10* cones. Similar to rods, the levels of several tubulin cytoskeleton-associated transcripts (including *Tubb5*, *Tubb4b*, and *Agtpbp1*) were also increased in *rd10* cones. We also observed a notable increase in *Capns1*, which encodes the regulatory calpain subunit, in *rd10* cones but not rods (Additional file 11). Calpain activity may be involved in photoreceptor degeneration in multiple RP models [30].

Several genes involved in phototransduction (*Gnat2*, *Opn1sw*, *Opn1mw*, *Guca1b*, *Pde6g*, *Pdc*) were downregulated in *rd10* cones prior to the onset of their degeneration (Fig. 3C; Additional file 11). This observation is in line with our previous report showing a transient downregulation of cone-specific genes early after light exposure, which primarily damages rods [38]. Interestingly, transcripts of the ‘rod-specific’ *Pde6g* gene were detected in cones, an observation already made by others in various mouse models [66–68]. Aside from genes required for phototransduction, rod degeneration significantly affected mitochondria in neighboring cones. As in degenerating rods, various mitochondrially encoded genes involved in oxidative phosphorylation were downregulated in *rd10* cones (Fig. 3C, Additional file 11). Among those were multiple subunits of the respiratory complex I (*mt-Nd1-5*, *Ndufaf5*, *Ndufa10*), *mt-Cytb*, all mitochondrially encoded subunits of the respiratory complex IV (*mt-Co1-3*), subunits 6 and 8 of the ATP synthase F_o_ region (*mt-Atp6*, *mt-Atp8*) as well as the nuclear-encoded hexokinase 2 (*Hk2*) (Additional file 11).

In general, we found an extensive overlap of differentially regulated genes between rods and cones. Among 332 statistically significant DE genes in cones, 118 were also DE among rod clusters, including *Egr1*, *Krt18*, *Nr4a1*, *Junb*, *Agtpbp1*, *Fgf2*, *Hspa1a*, *Hspa1b*, *Hk2*, and various mitochondrial genes (Additional file 11). Correlation analysis between *rd10* cones, wild-type cones, and rod clusters confirmed that *rd10* cones were transcriptomically more similar to degenerating rods than wild-type cones to wild-type rods (Fig. 3D).

### Expression levels for transcripts of interest in *rd10* retinas with increasing age

For additional validation of our single-cell transcriptomic findings, we determined the levels of selected transcripts with semi-quantitative real-time PCR (qPCR) in whole retinas of *rd10* and control mice at eight time points, from 2 weeks up to 6 months of age (Additional file 12: Fig. S4).

*Egr1* and *Agtpbp1* were selected as representative transcripts activated in damaged rods and cones. *Egr1* reached its peak expression in P21 and remained slightly above controls up to P49. This effect was statistically significant only in P21, in line with a transient activation of *Egr1* in rods, the most abundant cell type of the retina (Fig. 2D). Whereas *Agtpbp1* levels were similar between *rd10* and control retinas at P21, it was significantly upregulated at P28, a time point when most rods are in late stages of degeneration [6]. This correlated well with the strongly increased expression in *rd10* rods of cluster C03 that contained cells further along the degenerative process (Fig. 2D). Interestingly, *Agtpbp1* levels were significantly lower in *rd10* retinas compared to controls at P14, prior to the onset of rod degeneration. This may be linked to pre-degenerative outer segment defects in these retinas [69].

To test our findings regarding the decrease in mitochondrial transcripts, we analyzed *mt-Atp6*, *mt-Co2*, and *mt-Nd4* levels in the retina. While they were not strongly regulated when analyzed in whole retinas, we noted significantly lower values for all three genes in *rd10* retinas during the phase of strong rod degeneration (P21 for *mt-Atp6* and *mt-Co2*, P28 for *mt-Nd4*). No significant differences were detected in the expression of *Tfam*, a key mitochondrial transcriptional factor.

While droplet sequencing analyzes gene expression changes in distinct subpopulations of cells, qPCR averages expression levels over all cells in a tissue, which may explain differences between the two sets of data. Regardless, qPCR recapitulated the sequential upregulation pattern in *Egr1* and *Agtpbp1*, supporting the pseudotime analysis where the peak of *Egr1* expression was an early but transient event that preceded the peak of *Agtpbp1* expression (Fig. 2D). Since *Agtpbp1* was among the predicted target genes of EGR1 (Additional files 8 and 9) and EGR1 may act as a repressor of *Agtpbp1* expression (Additional file 10: Fig. S3), it is tempting to speculate that the late upregulation of *Agtpbp1* in *rd10* rods is directly connected to the downregulation of *Egr1* at this point in pseudotime (Fig. 2D).

### EGR1 constitutes an early response to degeneration in both rods and cones

**Fig. 4:**
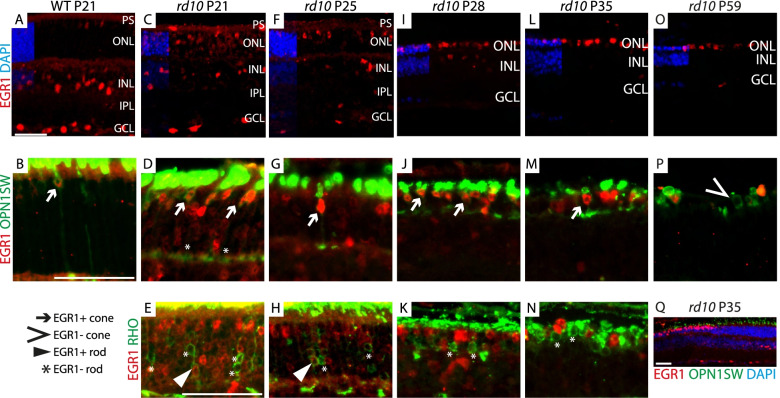
Localization of EGR1 protein in the degenerating *rd10* retina. Immunofluorescence against EGR1 (red) alone or in combination with OPN1SW (green) as a cone marker or RHO (green) as a marker for the perikarya of degenerating rods was performed in retinas of **A**, **B** wild-type controls, **C**–**E ***rd10* at P21, **F**–**H ***rd10* at P25, **I**–**K ***rd10* at P28, **L**–**N**, **Q ***rd10* at P35, and **O**, **P ***rd10* at P59. DAPI (blue) was used as a nuclear stain. Scale bars: 50 *μ*m. PS, photoreceptor segments; ONL, outer nuclear layer; INL, inner nuclear layer; IPL, inner plexiform layer; GCL, ganglion cell layer; WT, wild type

Based on our findings that *Egr1* mRNA was the most highly upregulated transcript in both early degenerating rods and in cones responding to primary rod degeneration, we hypothesized that EGR1 might be involved in an early stress response shared by both types of photoreceptors. To investigate the localization of EGR1 protein during degeneration, we combined immunostainings against EGR1 with short-wavelength opsin (OPN1SW) or rhodopsin (RHO) to discriminate between EGR1-expressing cones and rods, respectively (Fig. 4), and with glutamine synthetase (GS) to label Müller glia (Additional file 13: Fig. S5A-C).

In wild-type retinas, EGR1 immunoreactivity was confined mainly to the inner nuclear layer (INL) and ganglion cell layer (GCL), with few positive cells in the outer nuclear layer (ONL) (Fig. 4A). Co-immunostaining with OPN1SW and the location of the positive nuclei in the distal ONL identified these cells as cones (Fig. 4B, arrow). Müller glia, identified by GS staining, did not co-localize with the EGR1 signal in the INL (Additional file 13: Fig. S5A). Findings for wild-type neurons were in agreement with scRNAseq, which detected *Egr1* mRNA in few cones, subsets of bipolar and amacrine cells, and microglia (Additional file 14: Fig. S6). In contrast, the relatively high levels of *Egr1* mRNA detected in wild-type Müller glia did not correlate to immunoreactivity. The successful detection of EGR1 in some Müller glia in *rd10* retinas (Additional file 13: Fig. S5B, C) suggested a potential cell-type-specific regulation of EGR1 at the post-transcriptional level [70, 71]. EGR1 localization in wild-type retinas was comparable at all time points tested (Fig. 4A and Additional file 13: Fig. S5A for P21, S5D for P35).

At P21, *rd10* retinas displayed a major increase in the number of EGR1-immunopositive cells in the ONL compared to wild-type retinas (Fig. 4C). Since the EGR1 signal was present both in OPN1SW (Fig. 4D, arrows) and RHO (Fig. 4E, triangles) positive cells, this indicates that both rods and cones activated EGR1 in degenerating *rd10* retinas. However, some rods with an intense RHO signal in the perikaryon were only faintly positive for EGR1 immunoreactivity (Fig. 4E, asterisks). We additionally observed some Müller glia in *rd10* retinas at P21 that were EGR1-positive, including few with their nuclei shifted towards the ONL (Additional file 13: Fig. S5B, C; arrows). A similar pattern of EGR1 localization was observed in *rd10* retinas at P25 (Fig. 4F–H).

The pattern of EGR1 immunoreactivity in the ONL changed with progressing degeneration from P28 onward (Fig. 4I, L). Cones remained EGR1-positive (Fig. 4J, M; arrows), whereas many rods now lacked EGR1 signal (Fig. 4K, N; asterisks), in support of our transcriptomic data showing that *Egr1* expression in rods decreased with increasing degeneration. At P59, *rd10* retinas lost almost all rods [7], and the ONL mostly contained a single row of cone nuclei. While the number of cells with EGR1 immunoreactivity was generally low at late time points, several cones and few cells of the inner retina remained EGR1-positive (Fig. 4O, P). Some cone cells with no outer segments and OPN1SW staining only in the perikaryon had no EGR1 immunoreactivity (Fig. 4P, arrow). These cells were likely at advanced stages of secondary cone degeneration.

Overall, the number of EGR1-positive cells in the inner retina seemed to decrease with the progression of degeneration (Fig. 4C, F, I, L, O). The dependence of EGR1 localization on the degenerative state of the *rd10* retina was especially apparent at later time points such as P35, where we occasionally observed mildly affected peripheral areas with higher numbers of photoreceptors and better conserved outer segments, adjacent to regions with severe degeneration (Fig. 4Q). In mildly affected areas, the number of EGR1-positive cells was high in the inner retina and low in the ONL, whereas the pattern was largely reversed in the strongly affected regions (Fig. 4Q). This pattern might further bolster previous a hypothesis proposing that EGR1 expression is regulated by a complex communication between photoreceptors and inner retinal cells through their synaptic network [70].

## Discussion

Due to the heterogeneity of the retina and its cell-type-specific degenerative processes, high-resolution approaches such as scRNAseq are invaluable for mechanistic studies of inherited retinal degenerations. Using droplet sequencing, we identified two transcriptomically distinct stages of rod degeneration, characterized by the sequential activation of *Egr1* and *Cebpd*. Furthermore, we identified cone responses to primary rod cell death and showed that *Egr1* is also a part of early transcriptomic changes in cones, which include potential respiratory dysfunction.

### Rod transcriptomes at the peak of degeneration

In order to resolve the heterogeneity among degenerating rods, we performed trajectory analysis and defined a single pseudotemporal trajectory that originated from wild-type C01 cells, followed by *rd10* C02 cells, and ended at *rd10* C03 cells. Therefore, we hypothesized that C02 rods represented an early stage of degeneration, whereas C03 rods were further along in the same process.

#### Early events in rod degeneration

Hallmarks of early degenerative events in *rd10* rods included the activation of IEGs, changes in genes associated with water, ion and retinoid homeostasis as well as aerobic glycolysis, a potential induction of signaling events for cellular protection, and the downregulation of genes involved in phototransduction, potentially to save energy and/or prevent oxidative stress.

IEG activation constitutes the first response to a variety of intrinsic and extrinsic stimuli, including stressors. Many are transcription factors that orchestrate cellular responses by regulating downstream targets before they are rapidly inactivated [71]. *Egr1* was the most highly upregulated IEG early in rod degeneration (Additional file 5). *Egr1* is a downstream target of many signaling pathways [72], including those relevant for retinal neuroprotection [73, 74] and photoreceptor synaptic stability [48, 75, 76]. As such, *Egr1* levels were found to be differentially regulated by diverse forms of retinal stress and injury including photoreceptor degeneration in *rd1* [15] and *rds* mice [16], in retinoschisis [77] and after optic nerve crush in rats [78]. In *rds* mice, *Egr1* was detected specifically in rods further supporting a general role for *Egr1* in the photoreceptor stress response. While *Egr1* expression was upregulated in all of the above models, it was reduced in mouse retinas after hypoxia [79] and in the Akita mouse model of diabetes [80]. Thus, regulation of *Egr1* may depend on the stimulus and, potentially, the cell types affected by the stress.

Our in silico analysis pointed to EGR1 as a potential regulator of genes involved in rod degeneration (Additional file 8), including *Gadd45b* and *Agtpbp1*, genes with steadily increasing expression levels with the progression of degeneration (Fig. 2D, Additional file 6). Downregulating *Egr1* in photoreceptor-like 661W cells in vitro led to the upregulation of these two transcripts. While counterintuitive at first sight, this result is in line with EGR1’s dual function as both activator and repressor of gene expression, sometimes acting in opposite ways on the same target gene depending on the cellular context [59]. While speculative, EGR1 might assume either activity in retinal cells according to their degenerative state. The late upregulation of *Agtpbp1* and *Gadd45b* (Fig. 2D, Additional file 6) in degenerating rods might thus be directly connected to the reduced expression of *Egr1* and thus attenuated repression of some target genes at this point in pseudotime. Together, our results highlight a potential role of EGR1 in the reaction of rods to stress. Importantly, *Egr1* was also the highest upregulated transcript in cones. However, while *Egr1* expression declined in rods along the trajectory and in whole retinas as degeneration progressed, it remained high in cones that persisted in the *rd10* retina for some time after primary rod death (see below).

The activation of IEGs accompanied the induction of transcripts linked to calcium (*Camk2g*, *Pcp4*) and apoptosis/neuroprotective signaling (*Gadd45b*, *Krt18*, *Fgf2*, *Stat3*) as well as to ion, water, and retinoid homeostasis (*Atp2b1*, *Aqp1*, *Abca4*, *Kcnb1*, *Atp2b1*) (Additional file 5). Changes in transcripts related to calcium signaling validated the degenerative state of the early *rd10* rods, as the *rd10* mutation induces a detrimental Ca^2+^ influx in affected cells [5]. Together, these transcripts point to the activation of defense mechanisms in early rod stress, potentially including the initiation of an anti-apoptotic response, suggested by the early upregulation of *Gadd45b* and *Krt18*, which have anti-apoptotic functions in other contexts [34, 35, 81].

Another hallmark of early rod degeneration was the downregulation of transcripts related to phototransduction (Fig. 2D, Additional file 5), potentially to reduce energy consumption and channel available resources towards survival. Such energy-saving strategies may be particularly important since glycolytic enzymes *Hk2* and *Eno1* were also downregulated in early degenerative rods (Additional file 5), indicating early changes in energy metabolism. In rod-specific *Hk2* knockout mice, the resulting metabolic deficiency led to increased oxidative phosphorylation and a decrease in photoreceptor-specific proteins [40]. However, in *rd10* rods, oxidative phosphorylation is likely affected with further degeneration (see below), making energy conservation crucial. Consistent with downregulated phototransduction, transcripts that regulate rod photoreceptor fate and expression of phototransduction genes (*Nrl* [82], *Nr2e3* [83]; Additional file 5) were also downregulated in early degenerative rods.

#### Late events in rod degeneration

The late phase of degeneration in rods was characterized by a shift of transcription factors from *Egr1* to *Cebpd*, the remodeling or maintenance of rod cell structures, and the modulation of respiratory functions.

While *Egr1* was the most prominently upregulated transcript in early degeneration, *Cebpd* may be involved in late degenerative events. In lipopolysaccharide-activated microglia in vitro, EGR1 interacts with the *Cebpd* promoter, suggesting a link between the two transcription factors [84]. The induction of *Cebpd* was previously detected in the *rd10* model [8] and light damage [41], and its upregulation is generally associated with neuroprotection [85–87] (reviewed in [88]). Since *Cebpd* mRNA was shown to remain high in *rd10* retinas even after photoreceptor degeneration was complete [8], CEBPD may act in other cells in advanced degeneration. In this respect, it is notable that CEBPD was shown to be crucial for axon regeneration in the peripheral nervous system [89]. However, the roles CEBPD may play in degenerating photoreceptors are yet to be characterized.

Neural remodeling in the inner retina is a hallmark of both human RP [90–92] and its mouse models [92, 93] including *rd10* [6, 94], where subtle changes in synaptic transmission are detectable as early as P18 [6]. Synapse-related transcripts (e.g., *Rock2*, *Agrn*, *Slc17a7* [94]) were differentially regulated in late degenerative *rd10* rods (Additional file 5). RhoA-ROCK signaling mediates photoreceptor axon retraction in retinal detachment [48, 75] and ROCK plays a pro-apoptotic role in the Royal College of Surgeons rat model of RP [47]. Agrin is a heparan sulfate proteoglycan present in developing chick [95] and mouse retinas [96] with functions in neurogenesis and synapses [97–99]. As EGR1 was shown to repress *Agrn* expression at the neuromuscular junction [100], the downregulation of EGR1 in the late phase of rod degeneration may contribute to synaptic changes through releasing *Agrn* from a repressed state.

Modulation of photoreceptor cellular structures in late degeneration may happen not only in synapses but also in the photoreceptor segments. The photoreceptor outer segment is responsible for phototransduction and is connected to the cell body through a connecting cilium formed by doublet microtubules. Upregulation of transcripts encoding for tubulin proteins and the tubulin deglutamylase NNA1 (*Agtpbp1*) during late degeneration may indicate structural alterations to the rod outer segment. Defects in rod outer segments were observed already prior to photoreceptor degeneration at around P15 in the *rd10* model [69]. The upregulation of the tubulin deglutamylase suggests that post-translational tubulin modifications may be affected in degenerating photoreceptors at P21; genetic defects in these modifications lead to photoreceptor degeneration [101, 102]. Whether tubulin dysregulation also plays a role in photoreceptor degeneration in RP, and whether restoring tubulin modifications may help to ameliorate the degenerative process, remains to be explored. Interestingly, transcripts for tubulins and AGTPBP1 were also upregulated in cones, suggesting that these cells may also remodel their structures or attempt to support the structural integrity of inner and outer segments in response to rod death.

Although metabolic changes started in the early phase (see above), significant downregulation of mitochondrial gene expression (*mt-Atp6*, *mt-Co1-3*, *mt-Cytb*, *mt-Nd1-5*; Fig. 2D, Additional file 5) in the late phase indicates that respiratory dysfunction develops mainly late during degeneration in *rd10* rods. Since this effect primarily involved mitochondrially encoded genes, impairments in mitochondrial DNA stability might be involved. This is supported by the protective effect of metformin in *rd10* [103] and *rd1* [104] mice. Metformin is an antihyperglycemic guanidine derivative [105] that activates AMP-activated protein kinase [106]. Applied to the *rd10* retina, metformin increased the copy number of mitochondrial DNA and induced expression of mitochondrial genes [103]. Thus, a decrease in mitochondrial activity and respiration may be one of the most significant hallmarks of the late stage of rod death.

### Cone responses to rod degeneration

Little is known about how primary rod degeneration affects cones, which do not express the causative mutant gene but nevertheless die in a secondary wave of degeneration. To rescue cone function and vision in patients, it is crucial to identify the early responses of cones to rod degeneration. Three major events may be of significance for cones at this stage. These include increased expression of EGR1, changes in signaling, and reduced mitochondrial gene expression.

Like in the early phase of degeneration in rods, *rd10* cones upregulated several IEGs including *Egr1*, *Nr4a1*, *Junb* and *Jund*, with *Egr1* as the highest upregulated transcript. Intriguingly, pseudotime analysis suggested that rods lose *Egr1* expression as their degeneration progresses in older *rd10* mice. Comparably, *rd10* cones exhibited persisting expression of EGR1 until secondary cone degeneration. Together, these findings indicate that EGR1 levels are elevated very early in stressed rods and cones, potentially to protect cellular integrity and function prior to the full activation of cell death mechanisms. Considering the diversity of roles played by EGR1 depending on cellular context [59], it will be of importance to define the function of EGR1 by artificially modulating its activity specifically in rods or cones to evaluate EGR1 as a treatment target to preserve photoreceptor function and vision.

Furthermore, we observed a striking dynamic regulation of EGR1 protein localization in degenerative retinas. While EGR1 was prominent in the inner retina of wild types, its distribution changed with retinal degeneration in *rd10* mice. While photoreceptors became EGR1-positive early in degeneration, cells in the inner retina concomitantly lost EGR1 immunoreactivity. This change of EGR1 localization was especially obvious in sections of *rd10* retinas where strongly degenerated regions neighbored less affected areas. The reduced expression of EGR1 in the inner retina of older *rd10* mice with advanced photoreceptor degeneration and loss of visual function may indicate that EGR1 expression in the inner retina may depend on signals from photoreceptors. Simon et al. previously showed that EGR1 protein levels increased in the inner retina shortly after the onset of light. Since the increase was independent of circadian gene expression [70], this suggests a communication between light-activated photoreceptors and cells of the INL. Our results corroborate and extend this finding, by indicating that healthy and functional photoreceptors need to be present to regulate EGR1 expression in the inner retina.

The second emerging pattern in *rd10* cones was the differential regulation of transcripts involved in signaling pathways, especially transforming growth factor-beta (TGF-beta). *Inhbb* and *Bambi* were two strongly upregulated transcripts that encode proteins associated with TGF-beta signaling. While BAMBI is a silencer of TGF-beta signaling [107], *Inhbb* is a member of the TGF-beta superfamily and encodes inhibin beta, which may have diverse, even opposing effects on downstream targets [108]. *Inhbb* mRNA is also found at higher levels in the P10 *Nrl-/-* retina, which is devoid of rods [109], and thus may have a role in cone development. However, the functions of either gene may have in degenerating photoreceptors is unclear.

Perhaps the most important finding related to *rd10* cones in our data was the downregulation of multiple transcripts involved in mitochondrial respiration. This is similar to the potential respiratory dysfunction we observed in *rd10* rods, and in line with previous work that describe metabolic dysfunction in cones at the onset of cone cell death, when rods have cleared from the retina [11]. Here, we show the reduction in the levels of mitochondria-related transcripts before cone death commences. Interestingly, *Nxnl1* was downregulated in early degenerating *rd10* rods, suggesting that cones may already experience impaired glucose metabolism [10] at P21. Metabolic changes in cones are further suggested by the decreased expression of *Hk2*, a key enzyme for aerobic glycolysis and required for survival of photoreceptors during aging [39, 40]. Together with the downregulation of mitochondrially encoded transcripts, our data indicate that cone metabolism is likely affected already before the completion of rod degeneration.

## Conclusions

In conclusion, our study provides insight into transcriptomic changes in rods and cones during the primary wave of rod degeneration in the *rd10* mouse, a model of human RP. Our results highlight EGR1 as a key protein during the early phase of the cell death process in rods, and of the response of cones to primary rod degeneration. We also provide evidence for mitochondrial dysregulation in rods and especially in cones as a reaction to rod loss. Mitochondrial and metabolic changes might have crucial implications for cone photoreceptor survival in retinas that degenerate due to mutations in rod-specific genes.

## Methods

### Mice

All animal experiments adhered to the standards of the ARVO Statement for the Use of Animals in Ophthalmic and Vision Research and the regulations of the Veterinary Office of Canton Zurich, Switzerland, under the animal experimentation license ZH141/2016 (single-cell sequencing), and ZH091/2019 (tissue collection). *rd10* mice on a C57BL/6 background (IMSR Cat# JAX:004297, RRID:IMSR_JAX:004297) from Jackson Laboratories (Bar Harbor, USA) and wild type C57BL/6 controls (IMSR Cat# CRL:027, RRID:IMSR_CRL:027), from Charles River (Wilmington, MA, USA) were maintained in the Laboratory Animal Services Center facilities of the University of Zürich, with a 14 h:10 h light to dark cycle and access to food and water ad libitum. Euthanasia was performed with CO_2_ exposure followed by cervical dislocation for ZH141/2016 animals, and with CO_2_ exposure followed by decapitation for ZH091/2019 animals. All samples were collected between 10 am and 12 am to prevent a potential influence by the circadian rhythm or time since light onset [70].

### Fundus imaging and optical coherence tomography

Pupils of the mice were dilated using drops of 1% Cyclogyl (Alcon Pharmaceuticals, Fribourg, Switzerland) and 5% Neosynephrine (Ursapharm Schweiz GmbH, Roggwil, Switzerland) 20 min before anesthesia with a subcutaneous injection of 51 μl ketamine (50 mg/kg, Parke-Davis) and 6 μl Rompun (2%, Bayer AG, Leverkusen, Germany) per 30 g body weight. Eyes were kept moist throughout the experiment with the topical application of 2% Methocel (OmniVision AG, Neuhausen, Switzerland). OCT and fundus imaging were carried out with the Micron IV system (Phoenix Research Labs, Pleasanton, CA, USA). Mice were kept on a heating pad throughout the procedure.

### Preparation of single-cell suspensions from mouse retinas

A modified version of the Worthington Papain Dissociation System (Worthington, Lakewood, NJ, USA) was used for tissue dissociation. All components of the dissociation system were equilibrated with 95% O2: 5% CO_2_ before use. Retinas were isolated from euthanized P21 mice through a slit in the cornea and kept in equilibrated 1X EBSS (Cat. No. 24010043, ThermoFisher Scientific, Waltham, MA, USA) on ice. After removing the vitreous with surgical forceps, both retinas from each mouse were combined and transferred to an equilibrated solution containing 4.76 units of papain (Worthington) and 23.8 units of DNase (Worthington) with 0.95 mM L-cysteine and 0.47 mM EDTA in 1X EBSS for 15 (*rd10*) to 20 minutes (wild type) of incubation in a 37 °C water bath. The incubation time was shortened for *rd10* retinas due to the increased fragility of the degenerative retina. After papain digestion, 30 units of DNase and 1.034 mg/ml ovomucoid + albumin (Worthington) were added to the retinal suspensions, which were then layered on top of a 10 mg/ml ovomucoid + albumin solution for a discontinuous density gradient and centrifuged at 70 × g for 6 min to pellet intact cells. Pelleted cells were resuspended in 1X PBS (-Ca^2+^, -Mg^2+^; ThermoFisher Scientific) + 1% BSA (ThermoFisher Scientific) for calcein-propidium iodide staining.

### Calcein - propidium iodide staining

Calcein-AM (Sigma-Aldrich, St. Louis, MO, USA) at a final concentration of 2 μM and propidium iodide (Sigma-Aldrich) at a final concentration of 3 μM were added to a 1:20 diluted aliquot of each retinal suspension for cell count and viability measurements using a Neubauer cell counting chamber (Brand GmbH, Wertheim, Germany). Five million cells were used for the subsequent methanol fixation.

### Methanol fixation

Methanol fixation was performed to prevent cell death and limit changes to single-cell transcriptomes during handling and transport to the sequencing facility. All steps of the methanol fixation were performed on ice. Five million cells were pelleted at 300 × g for 3 min and resuspended via dropwise addition of 1 ml 1X PBS (-Ca^2+^, -Mg^2+^; ThermoFisher Scientific) followed by gentle pipetting using a wide-bore P1000 pipette tip (ThermoFisher Scientific), centrifuged at 300 × *g* for 5 min at 4 °C, and resuspended again in 1 ml 1X PBS as described above. Centrifugation was repeated at 300 × *g* for 5 min at 4 °C. The pellet was resuspended in 100 ul 1X PBS (-Ca^2+^, -Mg^2+^), resuspended as described above with a wide-bore P200 tip. While gently vortexing the tube, 900 μL of chilled 100% methanol was added dropwise to cells. The cells were fixed on ice for 15 min, and fixation was confirmed using trypan blue staining (NanoEntek Inc, Seoul, Korea).

### Droplet sequencing

Methanol-fixed cells were pelleted at 3000 × *g* for 10 min at 4 °C and resuspended in 1X PBS with 1% BSA (ThermoFisher Scientific) and 0.5 U/μL RNasin (Promega, Madison, WI, USA) and filtered through a 40-μm Flowmi Cell Strainer (Sigma-Aldrich). From each sample generated from an individual mouse, 10,000 cells (maximum recovery) were loaded onto an individual lane of a 10X Genomics Chromium Single Cell microfluidics chip using the v3 chemistry (10X Genomics, Pleasanton, CA, USA). Libraries were pooled and sequenced on an Illumina NovaSeq 6000 platform (Illumina, San Diego, CA, USA) at the Functional Genomics Center of the University of Zurich/ETH Zurich.

### Bioinformatic analysis of single-cell RNA sequencing data

Sequencing raw data was converted to FASTQ files, mapped to the GRCm38.p5 genome assembly, and used to build a cell-gene matrix in the Cell Ranger pipeline. The combined gene expression matrix generated in CellRanger, which contained data from all samples, was analyzed using the R package Seurat v3.1. Quality control was performed in multiple steps. Initially, genes expressed in less than 3 cells, and cells with less than 200 transcripts were removed from the analysis. Based on plots of the number of unique genes vs. the number of transcripts for each cell, only cells with 200–6000 unique genes and 1000–20,000 transcripts were retained. Data were normalized with log normalization and a scale factor of 10.000. Five thousand most highly variable genes with 0.1–8 average expression and > 1 dispersion were selected for downstream analyses. Prior to dimensionality reduction, the data were scaled to ensure each gene had a mean expression of 0 and variance of 1 across all cells and differences based on total UMI count were regressed out. The first 50 principal components were used for the main analyses. Cells were embedded on a k-nearest neighbor graph, and clustering was performed with modularity optimization on a shared nearest neighbor graph with a resolution parameter of 0.8. tSNE was used for visualizations. Cell cluster identity was assigned by the expression of known marker genes.

After clustering, additional filtering and cluster merging steps were applied to two specific clusters (Additional file 2: Fig. S2—cluster names containing “omitted”). We noted the presence of a small rod cell cluster composed primarily of wild-type rods. These cells had lower levels of total features per cell (Additional file 2: Fig. S2B) and transcripts per cell (Additional file 2: Fig. S2C), as well as a higher percentage of mitochondrial transcripts (Additional file 2: Fig. S2D). Differential gene expression analysis between these rods and the remaining rod clusters (Additional file 2: Fig. S2E) revealed that these cells expressed higher levels of ribosomal transcripts. Together, these features indicated the low-quality of cells in this cluster as cytoplasmic RNA leaks out of the damaged cells, while mitochondrial and ribosomal transcripts remain part of the respective organelles [110]. A cluster co-expressing Müller glia and rod photoreceptor markers was also excluded (Additional file 2: Fig. S2F), as this likely represented a technical artifact from Müller glia in close contact with rod photoreceptors bringing transcripts from both cell types into a single droplet. Differential gene expression analysis was performed with Seurat using a Wilcoxon rank sum test and Bonferroni correction for multiple testing. Hemoglobin transcripts were removed from gene sets prior to plotting. Batch correction was not applied, as batch-like effects in the data (Fig. 1C) correlated well with known biological variability between degenerating and wild-type rods. For the rod cell subset, variable feature detection (first 3000) and PCA (first 12 dimensions) were repeated prior to trajectory analysis. For the cone cell subset, data was renormalized and rescaled prior to plotting.

### Removal of doublets based on marker gene co-expression

Doublet removal based on the number of UMIs was performed as described above. For filtering doublets based on marker gene co-expression, the code for a previously published algorithm [23] was kindly provided by Jie Wang (Johns Hopkins University). Briefly, this approach identifies marker genes with high statistical power to define specific clusters and removes cells that express such markers for multiple cluster types. For further details, see Hoang et al. [23].

### Pseudotime analysis for rod photoreceptors

The R package Slingshot [31] was used to determine the pseudotime trajectory among rods, with the wild-type C01 cluster set as the starting cluster. A transcript list for further analysis was constructed by performing differential gene expression analysis as described above on clusters adjacent in pseudotime (C01 vs C02, and C02 vs C03) and including all genes with an adjusted *p*-value < 0.05 (Bonferroni correction). Then, rods were separated into 20 initial bins along pseudotime, and any adjacent bins with < 100 cells were merged, resulting in 16 bins. The average expression of each significant DE transcript in each pseudotime was obtained with Seurat [26]. The binned pseudotime matrix was then scaled for each gene, and the scaled expression values were plotted using the ComplexHeatmap package + [32], with *k* = 4 for k-means clustering. Heatmap colors were generated with the viridis palette with the R package circlize [111].

The expression profiles of all detected transcripts among rod photoreceptors were also fit to pseudotime values for each cell using the R package tradeSeq [56], which fits a generalized additive model for each gene. The associationTest function in the tradeSeq package was used to perform a Wald test to evaluate the null hypothesis that gene expression does not change along pseudotime. *P*-values were adjusted with Bonferroni correction.

### In silico position weight matrix (PWM) analysis

Lists of differentially expressed genes with adjusted *p*-value < 0.05 were provided as input to Enrichr (https://maayanlab.cloud/Enrichr/#) [112] to analyze the presence of transcription factor binding sites in the promoter regions (− 2000 to + 500 base pairs of the transcription start site) of these genes using the PWMs found in the TRANSFAC and JASPAR databases of transcription factors. The combined score, which is corrected for the statistical bias to the differences among numbers of potential targets of transcription factors [57, 112], was used to assess the results.

### Correlation analysis for *rd10* and wild-type cone photoreceptors

Rod clusters C00, C01, C02 and the cone cluster C04 were isolated from the main dataset, and the cone cluster C04 was separated into *rd10* and wild-type cones. The resulting five groups were used for the correlation analysis as follows: the average expression of 5000 most variable genes determined during the analysis of the main dataset was calculated for each group, and Spearman’s correlation coefficient was calculated using the average expression values with the R function *cor*.

### Cell culture and RNA interference

661W cells (RRID:CVCL_6240) were a kind gift from Dr. Muayyad al-Ubaidi [113]. Cells were cultured in high-glucose Dulbecco’s modified Eagle’s medium (DMEM) (Sigma-Aldrich) with 10% fetal bovine serum (FBS) and 100 U/ml penicillin and 100 μg/ml streptomycin (ThermoFisher) and maintained in a 37 °C humid incubator with 5% CO_2_. For siRNA transfection, 20,000 cells were seeded into each well of a 24-well plate (TPP, Trasadingen, Switzerland) 1 day prior. Transfections of siEgr1 (SMARTPool siGENOME Mouse Egr1 siRNA M-040286-01, Horizon Discovery Ltd., Waterbeach, UK) and a control siRNA (AllStars Negative Control siRNA, Qiagen, Hilden, Germany) were performed with Lipofectamine RNAiMAX reagent, according to kit instructions (ThermoFisher). Cells were collected 24 h after transfection for RNA extraction according to RNA extraction kit instructions (Macherey-Nagel NucleoSpin RNA kit, Oensingen, Switzerland).

### RNA extraction, cDNA synthesis, and semi-quantitative real-time PCR

Retinas were isolated from euthanized mice through a slit in the cornea, and snap-frozen in liquid nitrogen. Total RNA was isolated with a commercial, column-based RNA isolation kit with on-column DNaseI treatment (Macherey-Nagel NucleoSpin RNA kit). cDNA synthesis was carried out with oligo-(d)T selection and M-MLV reverse transcriptase (Promega). For semi-quantitative real-time PCR, 10 ng cDNA was used alongside primers targeting the gene of interest and the PowerUp SYBR Green Master Mix (ThermoFisher Scientific) in the ABI QuantStudio 3 system (ThermoFisher Scientific). Primers (Additional file 15) were designed, whenever possible, to span intron sequences or exon-exon boundaries to avoid genomic DNA amplification and tested for specificity and efficiency before experiments. *mt-Atp6* and *mt-Co2* primers were previously used in [114]. *mt-Nd4* primers were previously used in [115]. Beta-actin (*Actb*) was used as the reference housekeeping gene. Data analysis was carried out with the R statistical programming language, using the 2^-ddCt method [116]. For real-time PCR on retinal tissue, 3 biological replicates (individual mice from which retinas were isolated) were used per time point per strain. Two-way ANOVA (for effect of genotype and age) with Tukey’s range test was carried out on the resulting relative expression values with the R package rstatix. For real-time PCR on 661W cells, three replicate wells were transfected with the same siRNA (or left untreated) in each of the three individual experiments. Each experiment’s mean was used to calculate the mean of means and standard error of the mean. One-way ANOVA with Tukey’s range test was carried out on the three experimental means to determine the statistical significance of the results.

### Immunofluorescence on retinal cryosections

Eyes were marked dorsally via cauterization, and 12 μm sections were prepared as previously described [117]. Sections were blocked either in blocking solution (PB-Salt (0.1 M phosphate buffer with 0.8% NaCl and 0.02% KCl) with 0.3% Triton-X (Sigma-Aldrich) and 3% goat serum (Sigma-Aldrich) or 2% horse serum (Sigma-Aldrich)), followed by primary antibody incubation overnight at 4 °C. After three washes with PB-Salt, secondary antibody incubation was performed for an hour at room temperature. Nuclear counterstaining was performed with 4′,6-diamidine-2′-phenylindole di-hydrochloride (DAPI, Thermo Fisher Scientific). Sections were mounted using Mowiol (Sigma-Aldrich) and imaged with a Leica DMI6000B inverted fluorescent microscope (Leica Microsystems, Wetzlar, Germany). Primary and secondary antibodies used in the paper are listed in Additional file 16. Z-stacks were merged with max projection in ImageJ. Images were optimized for histogram minimum and maximum values using Adobe Photoshop.

## Supplementary Information

**Additional file 1: Figure S1.** Validation of the degenerative state of *rd10* retinas at P21. Fundus images (left panels) and OCT scans (right panels) of (A) *rd10* mouse 1, (B) *rd10* mouse 2, and (C) a wild-type mouse at P21. Mice were littermates of *rd10* and wild-type mice used for scRNAseq. Red lines in fundus images mark the location of OCT scans. The vertical black bars on OCT images mark the outer retina (outer plexiform layer to photoreceptor segments). Scale bars: 100 *μ*m.

**Additional file 2: Figure S2.** Additional quality control measures on the single-cell transcriptomic dataset. (A) Two-dimensional tSNE plot of the whole dataset before low-quality clusters were filtered. Filtered clusters have the tag “: Omitted” and are labeled with “M” (Müller glia-rods) and “R” (Rods) in the tSNE plot. (B-D) Quality control measures for the omitted rod cluster, compared to the three retained rod clusters C01, C02 and C03. The omitted cluster has lower numbers of total features (B) and transcripts (C) but a higher percentage of mitochondrial transcripts (“mt-genes”) per cell (D). (E) Differential gene expression analysis between omitted and retained rod clusters shows that omitted rods have higher levels of ribosomal transcripts. (F) Differential gene expression analysis between omitted and retained Müller glia cluster shows that omitted Müller glia are positive for rod cell markers (e.g., *Sag*, *Rho*) and express lower levels of Müller glia markers (e.g., *Dkk3*).

**Additional file 3.** Marker gene analysis for the single-cell transcriptomic dataset. To determine which transcripts distinguish each cluster in the dataset for cluster annotation, differential gene expression analysis between each cluster and the remainder of the dataset was performed with the Seurat package.

**Additional file 4. **Early differential gene expression analysis in rods. Wild type rod cluster C01 and *rd10* rod cluster C02, which were adjacent in the pseudotime analysis, were compared with differential gene expression analysis to determine transcriptomic differences that occurred with increasing pseudotime values. Avg_logFC values larger than 0 indicate that the gene is upregulated in C02 compared to C01.

**Additional file 5. **Late differential gene expression analysis in rods. *rd10* rod cluster C02 and *rd10* rod cluster C03, which were adjacent in the pseudotime analysis, were compared with differential gene expression analysis to determine transcriptomic differences that occurred with increasing pseudotime values. Avg_logFC values larger than 0 indicate that the gene is upregulated in C03 compared to C02.

**Additional file 6.** k-means clustering of significantly DE genes among rod clusters according to expression changes along increasing (binned) pseudotime. Rods were grouped into 16 bins along increasing pseudotime. Expression values for all significantly DE transcripts were averaged in each bin, scaled, and the values were used for k-means clustering (k=4) to cluster transcripts into groups of similar behaviour in pseudotime (and thus progressing degeneration). DE: differentially expressed.

**Additional file 7.** Association of gene expression to pseudotime values in the rod dataset. To determine which transcripts were associated with pseudotime, we performed a Wald test using the tradeSeq package (associationTest, no log2 fold-change threshold). The expression pattern of each gene in the entire dataset is modeled along pseudotime, and the Wald statistic is calculated to indicate whether the gene is associated with pseudotime. DE: differentially expressed.

**Additional file 8. **In silico position weight matrix analysis on transcripts differentially expressed in early degeneration. To determine which transcription factors might be associated with significantly differentially expressed transcripts identified in the early differential gene expression analysis (Additional file 2), we used Enrichr to perform an in silico search for sequence motifs representing transcription factor binding sites in the promoter region (-2000 to + 500 bp of the transcriptional start site) of these genes. The Overlap column refers to the number of genes in the input list (differentially regulated genes; numerator) compared to all genes in the database (denominator) that contain the transcription factor binding motif. P-values were calculated with Fisher’s exact test, and adjusted with the Benjamini-Hochberg method. The Combined Score is log(*p*-value)*z-score, where z-score is the deviation from the expected rank calculated from many random gene lists [57, 112].

**Additional file 9. **In silico position weight matrix analysis on transcripts differentially expressed in late degeneration. To determine which transcription factors might be associated with significantly differentially expressed transcripts identified in the late differential gene expression analysis (Additional file 3), we used Enrichr to perform an in silico search for sequence motifs representing transcription factor binding sites in the promoter region (-2000 to + 500 bp of the transcriptional start site) of these genes. The Overlap column refers to the number of genes in the input list (differentially regulated genes; numerator) compared to all genes in the database (denominator) that contain the transcription factor binding motif. P-values were calculated with Fisher’s exact test, and adjusted with the Benjamini-Hochberg method. The Combined Score is log(*p*-value)*z-score, where z-score is the deviation from the expected rank calculated from many random gene lists [57, 112].

**Additional file 10: Figure S3.** Expression levels of potential target genes after downregulation of *Egr1* in vitro. Expression levels of *Egr1*, potential EGR1-target genes (*Agtpbp1*, *Gadd45b*) and a non-target gene (*Apoe*) were determined in 661W cells 24 hours after transfection of an siRNA against *Egr1* (“siEgr1”, blue) or control siRNA (“siCtrl”, green), or in untransfected controls (“No TF”, red). Shown are the mean of three independent experiments (diamonds) ± standard error of the mean (error bars). Each individual point refers to the mean of an independent experiment. *: *p* < 0.05; one-way ANOVA with Tukey’s range test. TF: transfection.

**Additional file 11. **Differential gene expression analysis among *rd10* and wild-type cones in cluster C04. Differential gene expression analysis was performed to determine transcriptomic changes in *rd10* cones compared to wild types at P21 . All transcripts were additionally checked for significant differential expression in early rod degeneration (Additional file 2) or late rod degeneration (Additional file 3). Avg_logFC values larger than 0 indicate that the gene is upregulated in *rd10* cones compared to wild types. DE: differentially expressed.

**Additional file 12: Figure S4.** Relative expression levels of selected genes in *rd10* and wild-type retinas. Expression levels of selected genes were determined in retinas of *rd10* (blue) and wild-type (red) mice at various ages, relative to the average of C57BL/6 P21 retinas. Shown are boxplots with individual data points. *N*=3 per time point per strain. *: *p* < 0.05, **: *p* < 0.01, ***: *p* < 0.001, ****: *p* < 0.0001; two-way ANOVA with Tukey’s range test.

**Additional file 13: Figure S5.** Additional stainings for EGR1 in *rd10* and wild-type retinas. Co-immunofluorescence for EGR1 (red) and GS (green) as a Müller glia marker was performed in retinas of (A) wild-type control at P21 or (B-C) *rd10* mice at P21. (D) Co-immunofluorescence for (red) and OPN1SW (green) as a cone marker in a retinal section of a wild-type control mouse at P35. Arrows point to EGR1-positive Müller glia nuclei close to the outer rim of the INL. The arrowhead indicates an EGR1-positive Müller glia nucleus at its expected position. Scale bars: 50 *μ*m. PS: Photoreceptor segments. ONL: outer nuclear layer. INL: inner nuclear layer. IPL: inner plexiform layer. GCL: ganglion cell layer.

**Additional file 14: Figure S6. ***Egr1* expression in the whole dataset. Two-dimensional UMAP plot of the whole dataset, with cells colored according to their normalized expression levels of *Egr1*. For cluster identities, see B. (B) Heatmap of average normalized expression levels of *Egr1* within each cluster, split by genotype. Cluster 28 is composed only of *rd10* cells. wt: wild type.

**Additional file 15. **Primer sequences for quantitative PCR. *mt-Co2* and *mt-Atp6* primers are from Wall et al. 2015 [102]. *mt-Nd4* primers are from El-Merhie et al. 2017 [103].

**Additional file 16.** Antibodies used for immunofluorescence.

## Data Availability

The scRNA-seq dataset supporting the conclusions of this article is available in the Gene Expression Omnibus repository (GSE183206).
