# Supplementary figures and images for: Single-cell RNA sequencing of the retina in a model of retinitis pigmentosa reveals early responses to degeneration in rods and cones
Source: BMC Biol. 2022 Apr 12;20:86. doi: 10.1186/s12915-022-01280-9 (PMC9006580; doi:10.1186/s12915-022-01280-9)

*rd10* mouse 1

A

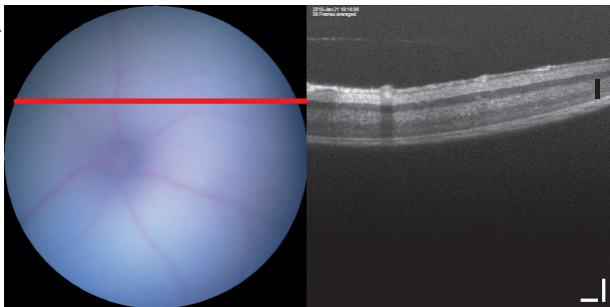

*rd10* mouse 2

B

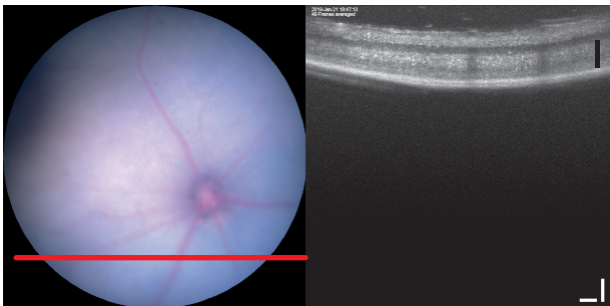

wild type

C

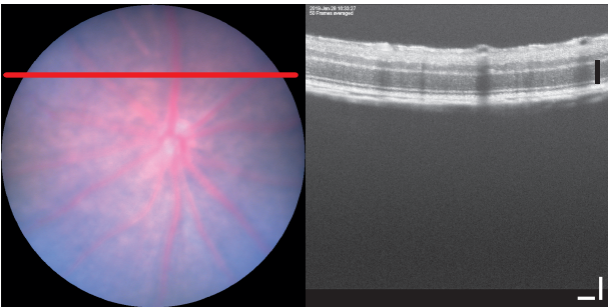

Supplement: Supplementary file 1 — Additional file 1: Figure S1. Validation of the degenerative state of rd10 retinas at P21. Fundus images (left panels) and OCT scans (right panels) of (A) rd10 mouse 1, (B) rd10 mouse 2, and (C) a wild-type mouse at P21. Mice were littermates of rd10 and wild-type mice used for scRNAseq. Red lines in fundus images mark the location of OCT scans. The vertical black bars on OCT images mark the outer retina (outer plexiform layer to photoreceptor segments). Scale bars: 100 μm. [file 12915_2022_1280_MOESM1_ESM.pdf]

Relative expression

*Egr1*

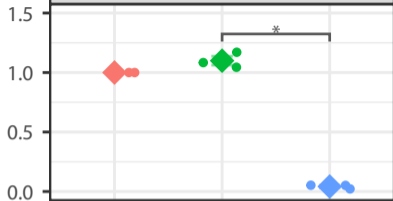

*Apoe*

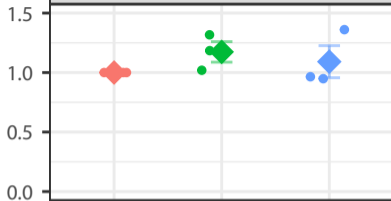

*Gadd45b*

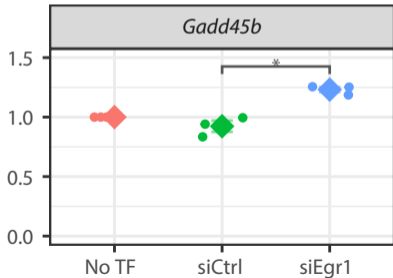

*Agtpbp1*

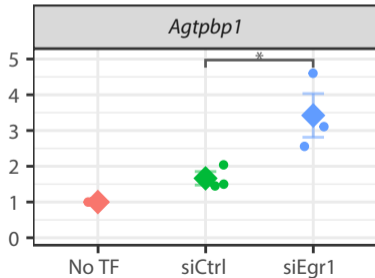

siRNA

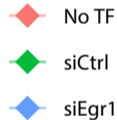

siRNA

Supplement: Supplementary file 10 — Additional file 10: Figure S3. Expression levels of potential target genes after downregulation of Egr1 in vitro. Expression levels of Egr1, potential EGR1-target genes (Agtpbp1, Gadd45b) and a non-target gene (Apoe) were determined in 661W cells 24 hours after transfection of an siRNA against Egr1 (“siEgr1”, blue) or control siRNA (“siCtrl”, green), or in untransfected controls (“No TF”, red). Shown are the mean of three independent experiments (diamonds) ± standard error of the mean (error bars). Each individual point refers to the mean of an independent experiment. *: p < 0.05; one-way ANOVA with Tukey’s range test. TF: transfection. [file 12915_2022_1280_MOESM10_ESM.pdf]

Genotype 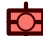 BL6 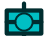 *rd10*

Relative expression

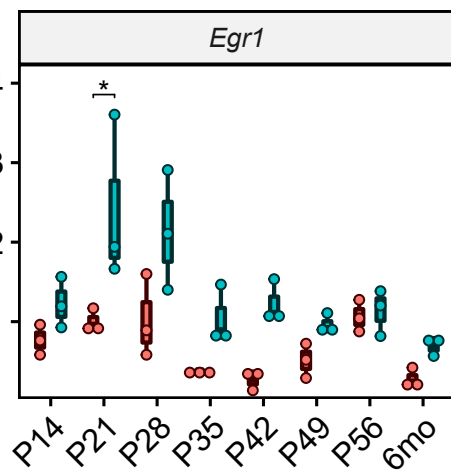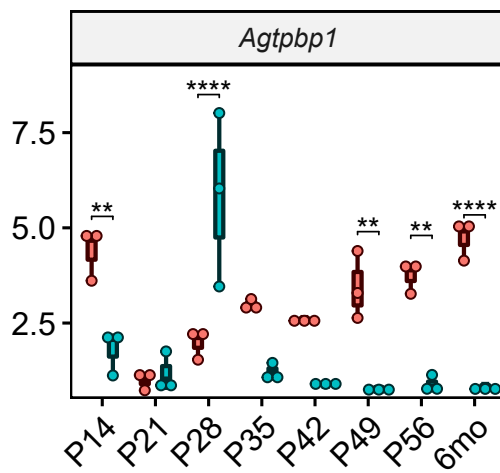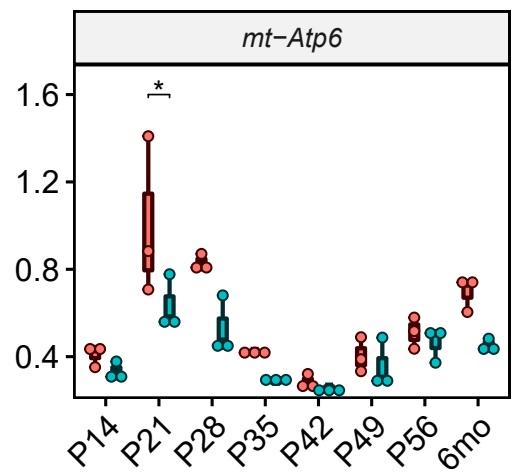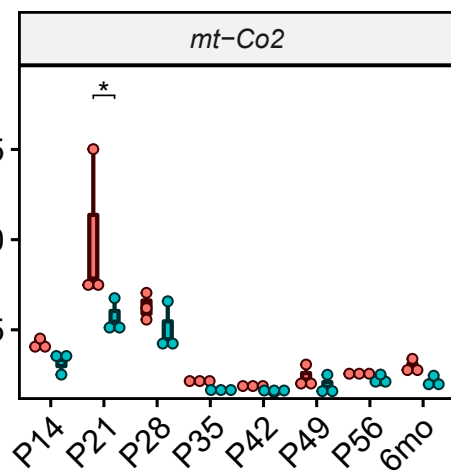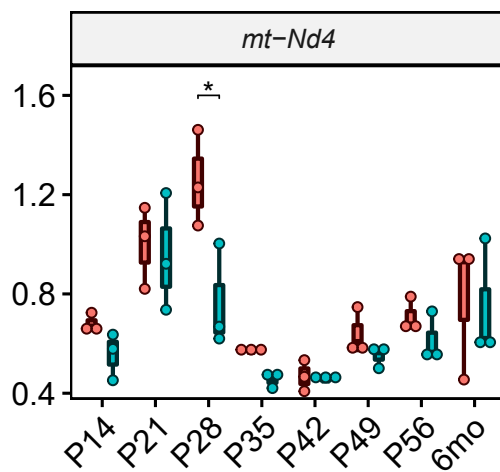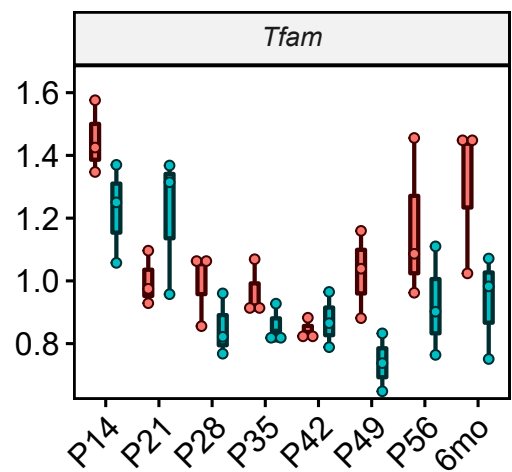

Age

Supplement: Supplementary file 12 — Additional file 12: Figure S4. Relative expression levels of selected genes in rd10 and wild-type retinas. Expression levels of selected genes were determined in retinas of rd10 (blue) and wild-type (red) mice at various ages, relative to the average of C57BL/6 P21 retinas. Shown are boxplots with individual data points. N=3 per time point per strain. *: p < 0.05, **: p < 0.01, ***: p < 0.001, ****: p < 0.0001; two-way ANOVA with Tukey’s range test. [file 12915_2022_1280_MOESM12_ESM.pdf]

WT P21

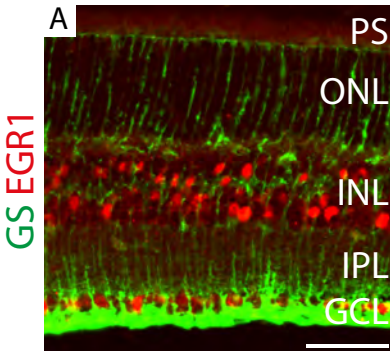

*rd10* P21

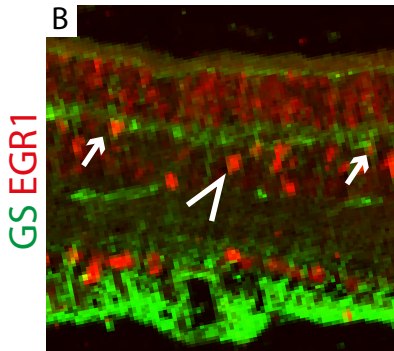

*rd10* P21

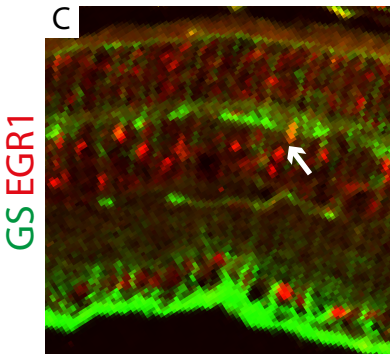

WT P35

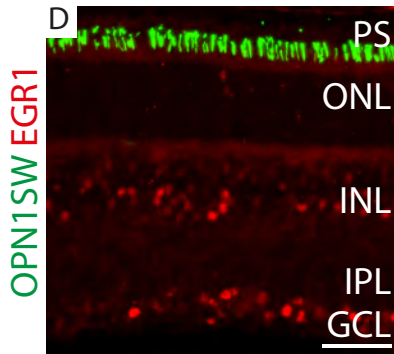

Supplement: Supplementary file 13 — Additional file 13: Figure S5. Additional stainings for EGR1 in rd10 and wild-type retinas. Co-immunofluorescence for EGR1 (red) and GS (green) as a Müller glia marker was performed in retinas of (A) wild-type control at P21 or (B-C) rd10 mice at P21. (D) Co-immunofluorescence for (red) and OPN1SW (green) as a cone marker in a retinal section of a wild-type control mouse at P35. Arrows point to EGR1-positive Müller glia nuclei close to the outer rim of the INL. The arrowhead indicates an EGR1-positive Müller glia nucleus at its expected position. Scale bars: 50 μm. PS: Photoreceptor segments. ONL: outer nuclear layer. INL: inner nuclear layer. IPL: inner plexiform layer. GCL: ganglion cell layer. [file 12915_2022_1280_MOESM13_ESM.pdf]

**A*****Egr1***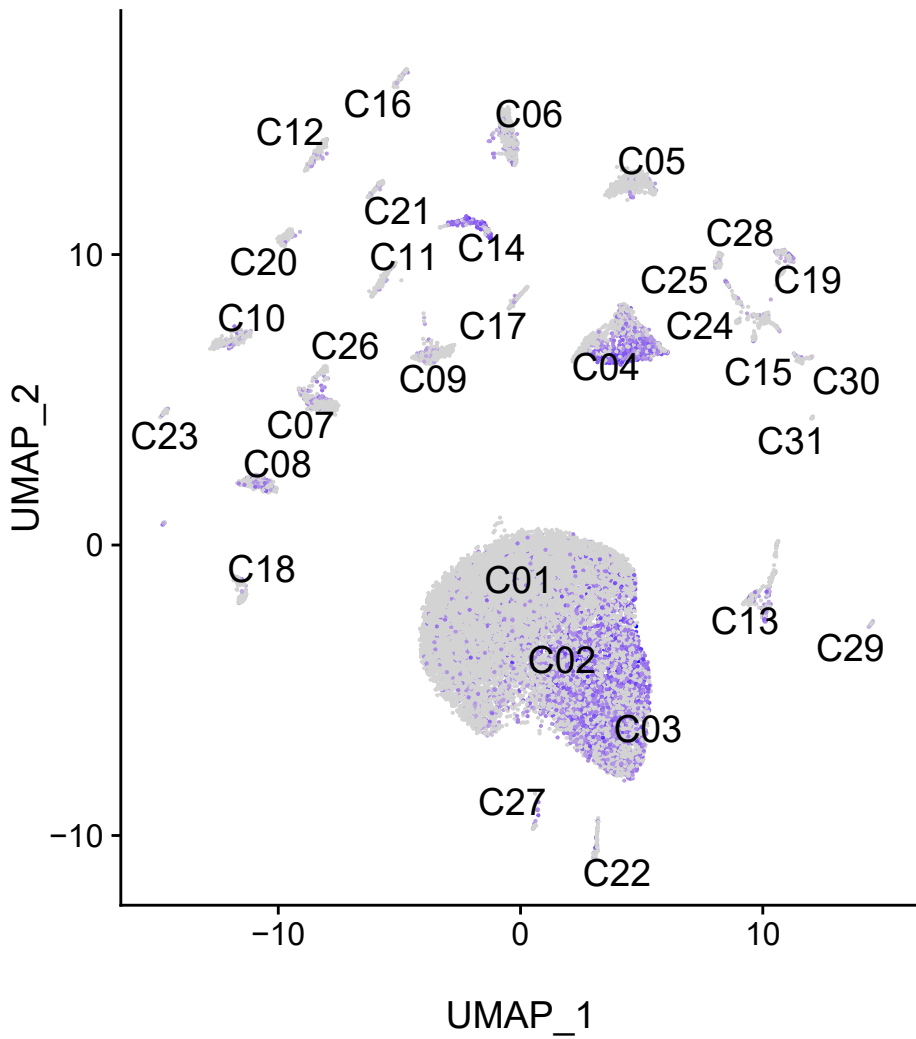**B**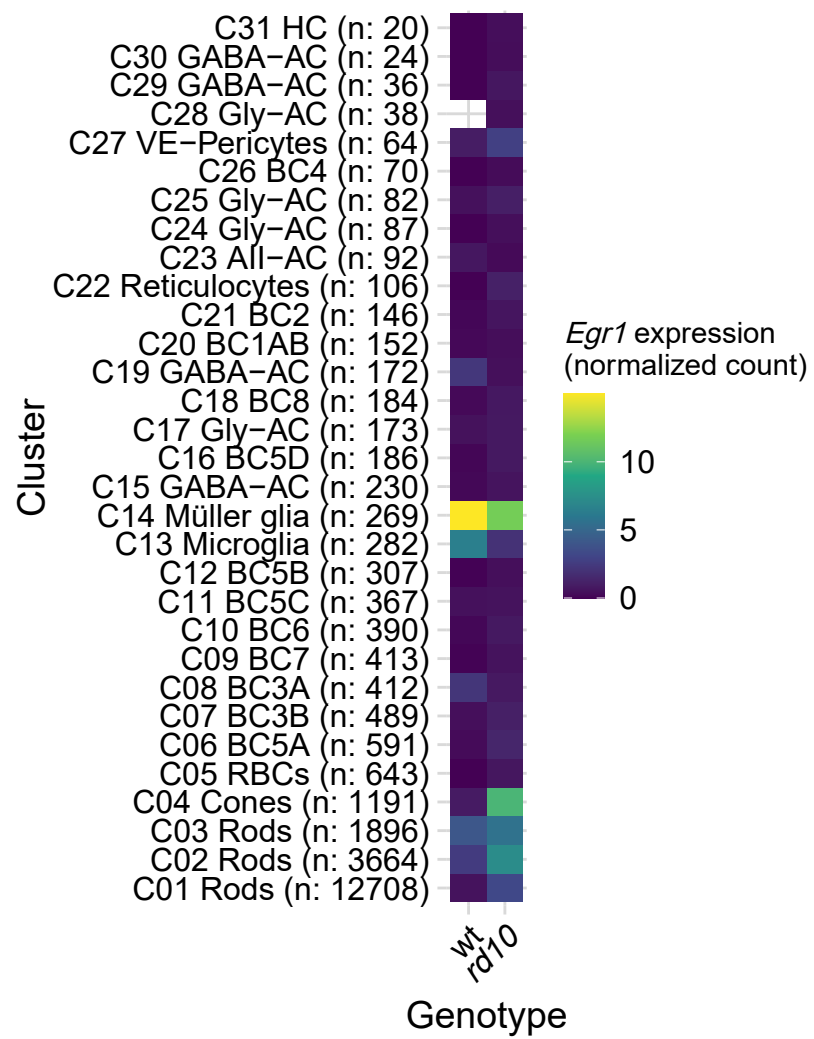

Supplement: Supplementary file 14 — Additional file 14: Figure S6. Egr1 expression in the whole dataset. Two-dimensional UMAP plot of the whole dataset, with cells colored according to their normalized expression levels of Egr1. For cluster identities, see B. (B) Heatmap of average normalized expression levels of Egr1 within each cluster, split by genotype. Cluster 28 is composed only of rd10 cells. wt: wild type. [file 12915_2022_1280_MOESM14_ESM.pdf]
