# Supplementary material for: Single-cell RNA sequencing of the retina in a model of retinitis pigmentosa reveals early responses to degeneration in rods and cones
Source: BMC Biol. 2022 Apr 12;20:86. doi: 10.1186/s12915-022-01280-9 (PMC9006580; doi:10.1186/s12915-022-01280-9)
Supplement: Supplementary file 2 — Additional file 2: Figure S2. Additional quality control measures on the single-cell transcriptomic dataset. (A) Two-dimensional tSNE plot of the whole dataset before low-quality clusters were filtered. Filtered clusters have the tag “: Omitted” and are labeled with “M” (Müller glia-rods) and “R” (Rods) in the tSNE plot. (B-D) Quality control measures for the omitted rod cluster, compared to the three retained rod clusters C01, C02 and C03. The omitted cluster has lower numbers of total features (B) and transcripts (C) but a higher percentage of mitochondrial transcripts (“mt-genes”) per cell (D). (E) Differential gene expression analysis between omitted and retained rod clusters shows that omitted rods have higher levels of ribosomal transcripts. (F) Differential gene expression analysis between omitted and retained Müller glia cluster shows that omitted Müller glia are positive for rod cell markers (e.g., Sag, Rho) and express lower levels of Müller glia markers (e.g., Dkk3). [file 12915_2022_1280_MOESM2_ESM.pdf]

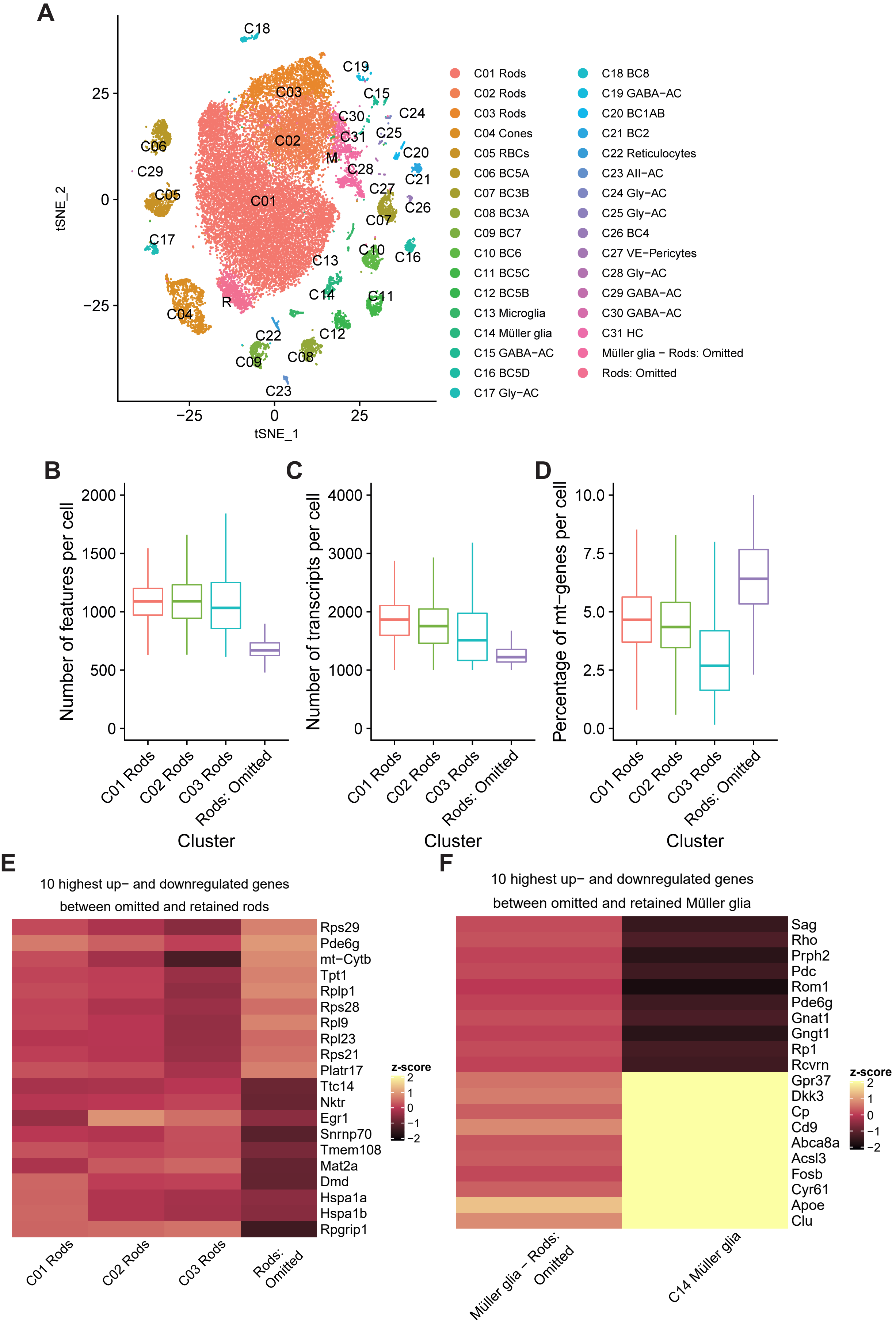
A
tSNE_1
tSNE_2
C01 Rods
C02 Rods
C03 Rods
C04 Cones
C05 RBCs
C06 BC5A
C07 BC3B
C08 BC3A
C09 BC7
C10 BC6
C11 BC5C
C12 BC5B
C13 Microglia
C14 Müller glia
C15 GABA−AC
C16 BC5D
C17 Gly−AC
C18 BC8
C19 GABA−AC
C20 BC1AB
C21 BC2
C22 Reticulocytes
C23 AII−AC
C24 Gly−AC
C25 Gly−AC
C26 BC4
C27 VE−Pericytes
C28 Gly−AC
C29 GABA−AC
C30 GABA−AC
C31 HC
Müller glia − Rods: Omitted
Rods: Omitted
B
Number of features per cell
C
Number of transcripts per cell
D
Percentage of mt−genes per cell
Cluster
E
10 highest up− and downregulated genes between omitted and retained rods
Rps29
Pde6g
mt−Cytb
Tpt1
Rplp1
Rps28
Rpl9
Rpl23
Rps21
Platr17
Ttc14
Nktr
Egr1
Snrnp70
Tmem108
Mat2a
Dmd
Hspa1a
Hspa1b
Rpgrip1
z-score
F
10 highest up− and downregulated genes between omitted and retained Müller glia
Sag
Rho
Prph2
Pdc
Rom1
Pde6g
Gnat1
Gngt1
Rp1
Rcvrn
Gpr37
Dkk3
Cp
Cd9
Abca8a
Acsl3
Fosb
Cyr61
Apoe
Clu
Müller glia – Rods: Omitted
C14 Müller glia
